# Supplementary material for: A key residue of the extracellular gate provides quality control contributing to ABCG substrate specificity
Source: Nat Commun. 2025 May 5;16:4177. doi: 10.1038/s41467-025-59518-3 (PMC12052975; doi:10.1038/s41467-025-59518-3)
Supplement: Supplementary file 1 — Supplementary Information [file 41467_2025_59518_MOESM1_ESM.pdf]

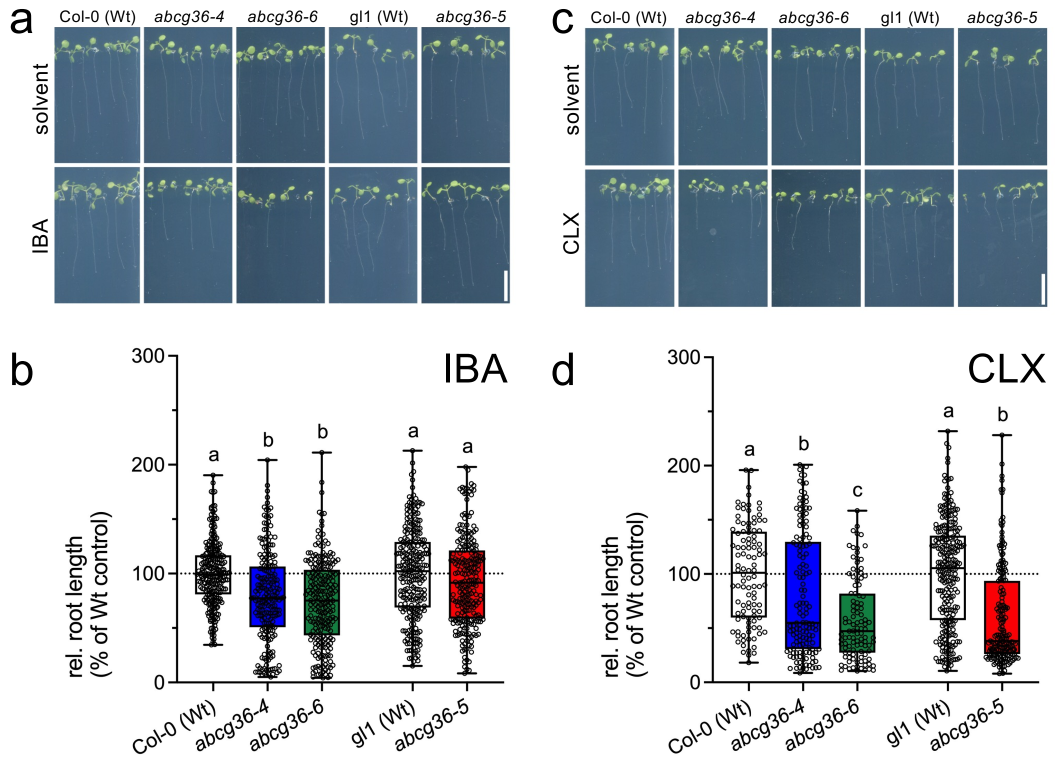

Supplementary Figure 1: In the *abcg36-5* allele, ABCG36-mediated IBA – but not camalexin - detoxification activity is retained

a-d. Relative root length of indicated Arabidopsis lines grown in the presence of 7.5 µM IBA **(a-b)** and 5 µg/mL CLX **(c-d)** for 12 days. Wt (Col-0 and gl1) growth is set to 100%. Results are mean values (±SE) of 4 independent experiments with 10-20 seedlings each. Different lowercase letters indicate that the means are significantly different (Ordinary one-way ANOVA with a post hoc Sidak's multiple comparison test, $p < 0.05$). Scale bar = 1 cm.

Data are presented as box-and-whisker plots, where median and 25th and 75th percentiles are represented by the box itself and the middle line, respectively; means are indicated by a "+". Source data are provided as a Source Data file.

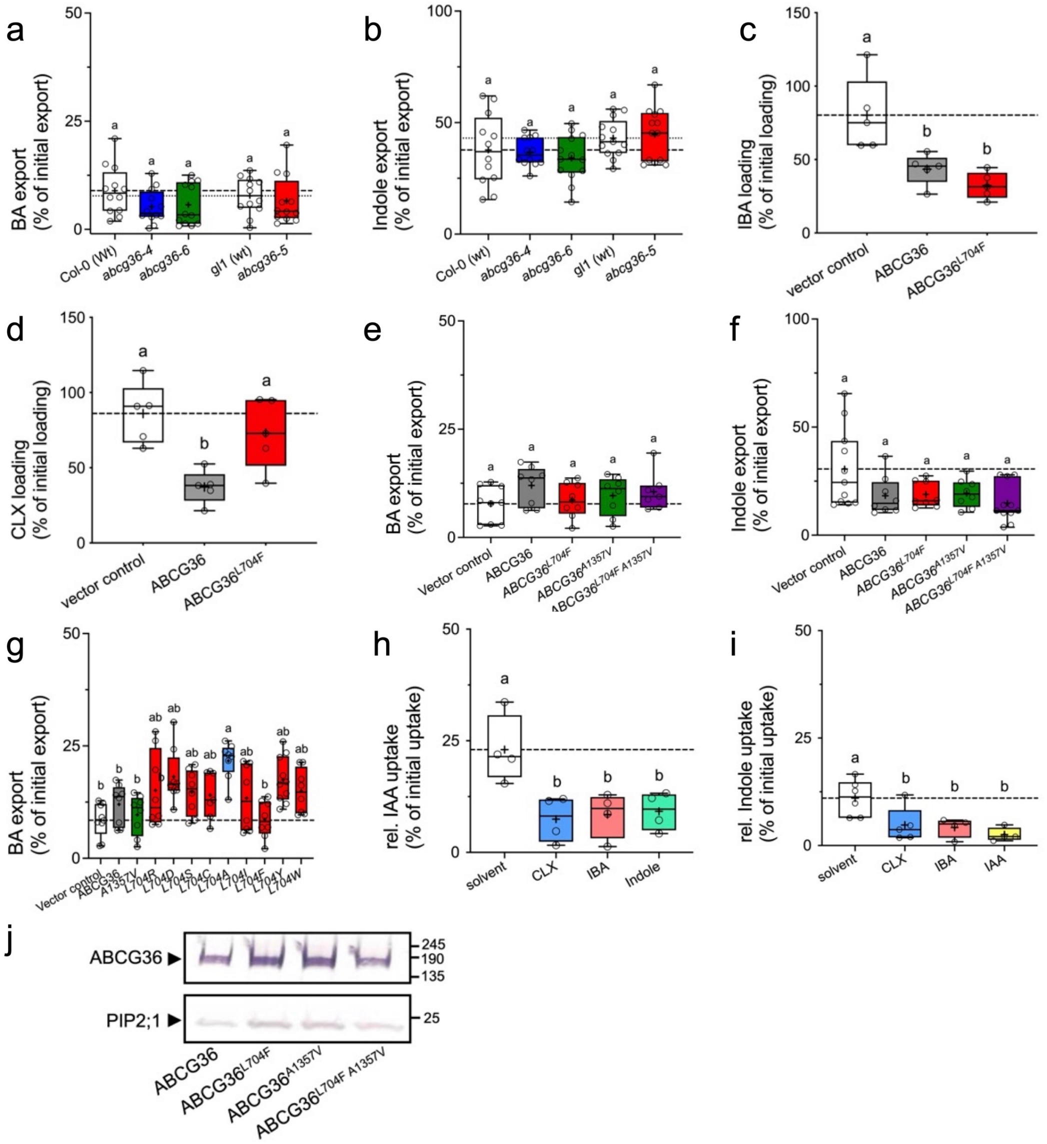

Supplementary Figure 2: Transport controls

a-b. BA (**a**) and indole (**b**) export from *Arabidopsis* protoplasts prepared from indicated *ABCG36* loss-of-function alleles. Significant differences (p < 0.05) of means ± SE (n ≥ 7 independent protoplast preparations) were determined using Brown-Forsythe and Welch ANOVA and are indicated by different lowercase letters.

c-d. IBA (**c**) and CLX (**d**) loading into *N. benthamiana* protoplasts after transfection with *ABCG36* or *ABCG36*^{L704F} Significant differences (p < 0.05) of means ± SE (n ≥ 7 independent protoplast preparations) were determined using Brown-Forsythe and Welch ANOVA and are indicated by different lowercase letters.

e-f. BA (**c**) and indole (d) export from *N. benthamiana* protoplasts after transfection with indicated mutant versions *ABCG36*. Significant differences (p < 0.05) of means ± SE (n ≥ 7 independent protoplast preparations) were determined using Brown-Forsythe and Welch ANOVA and are indicated by different lowercase letters.

g. BA export from *N. benthamiana* protoplasts after transfection with indicated mutant versions *ABCG36*. Significant differences (p < 0.05) of means ± SE (n ≥ 7 independent protoplast preparations) were determined using Brown-Forsythe and Welch ANOVA and are indicated by different lowercase letters. Additionally, means of mutant ABCG36 that are significantly different to Wt ABCG36 (grey fill) are indicated in red, while non-significant ones are in blue. ABCG36^{A1357V} (green) is included as a negative control.

h-i. Competition of IAA (f) and indole (g) uptake into microsomes prepared from ABCG36L704Y transfected tobacco leaves; concentration of indicated competitors was 100x higher than radiolabeled IAA and indole. Significant differences (p < 0.05) of means ± SE (n ≥ 4 independent transport experiments) were determined using One-way ANOVA and are indicated by different lowercase letters.

j. Western analyses of Wt and mutant versions of ABCG36-GFP (35S:PEN3-GFP) expressed in tobacco. Microsomes (each 10 ug protein) prepared from transfected tobacco were probed against anti-PDR8/ABCG36; the PM marker aquaporin (anti-PIP2;1) was used as a loading control.

Data are presented as box-and-whisker plots, where median and 25th and 75th percentiles are represented by the box itself and the middle line, respectively; means are indicated by a "+". Source data are provided as a Source Data file.

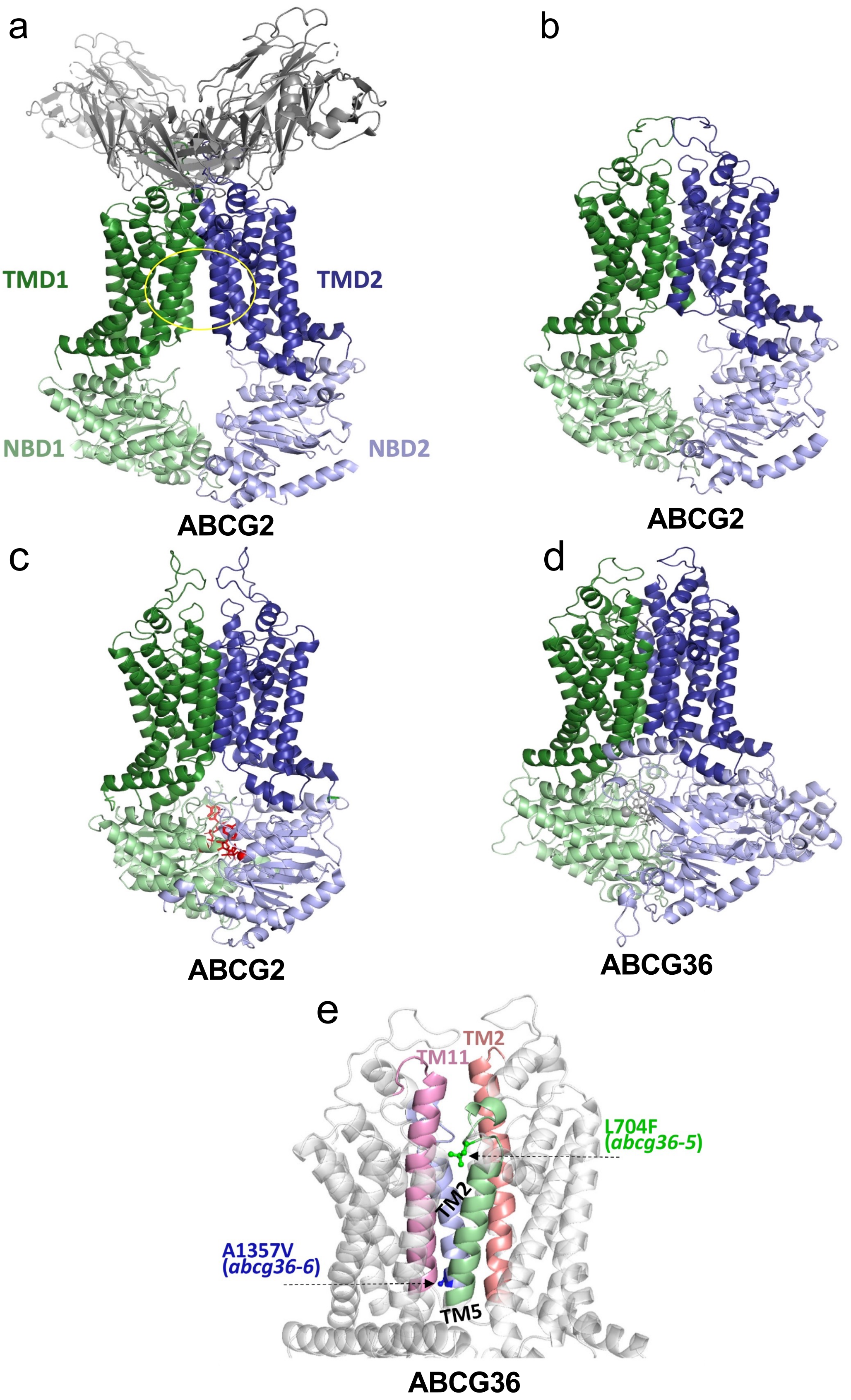

Supplementary Figure 3: Structural comparison of ABCG2 and ABCG36

a. The functional unit of ABC proteins consists of two transmembrane domains (TMD, dark green, and dark blue) and two nucleotide-binding domains (NBD, green and blue). The TMDs and NBDs of the human ABCG2 multidrug transporter are in an open conformation in the absence of ATP and with extracellular stabilizing antibody (gray); the central binding pocket is indicated by a yellow circle (PDBID: 5nj3).

b. In an *apo* ABCG2 structure (PDBID: 6vxf), the NBDs are open but TMDs are closed.

c. In the presence of ATP both NBDs and TMDs are closed (PDBID: 6hbu).

d-e. The AlphaFold2-predicted ABCG36 structure exhibits a closed conformation. This structure was used in our study, since this structure is the closest to the transport-competent conformation (**d**). Location of employed ABCG36 alleles, *abcg36-5* (L704F) and *abcg36-6* (A1357V), respectively, as well as THs are illustrated (**e**).

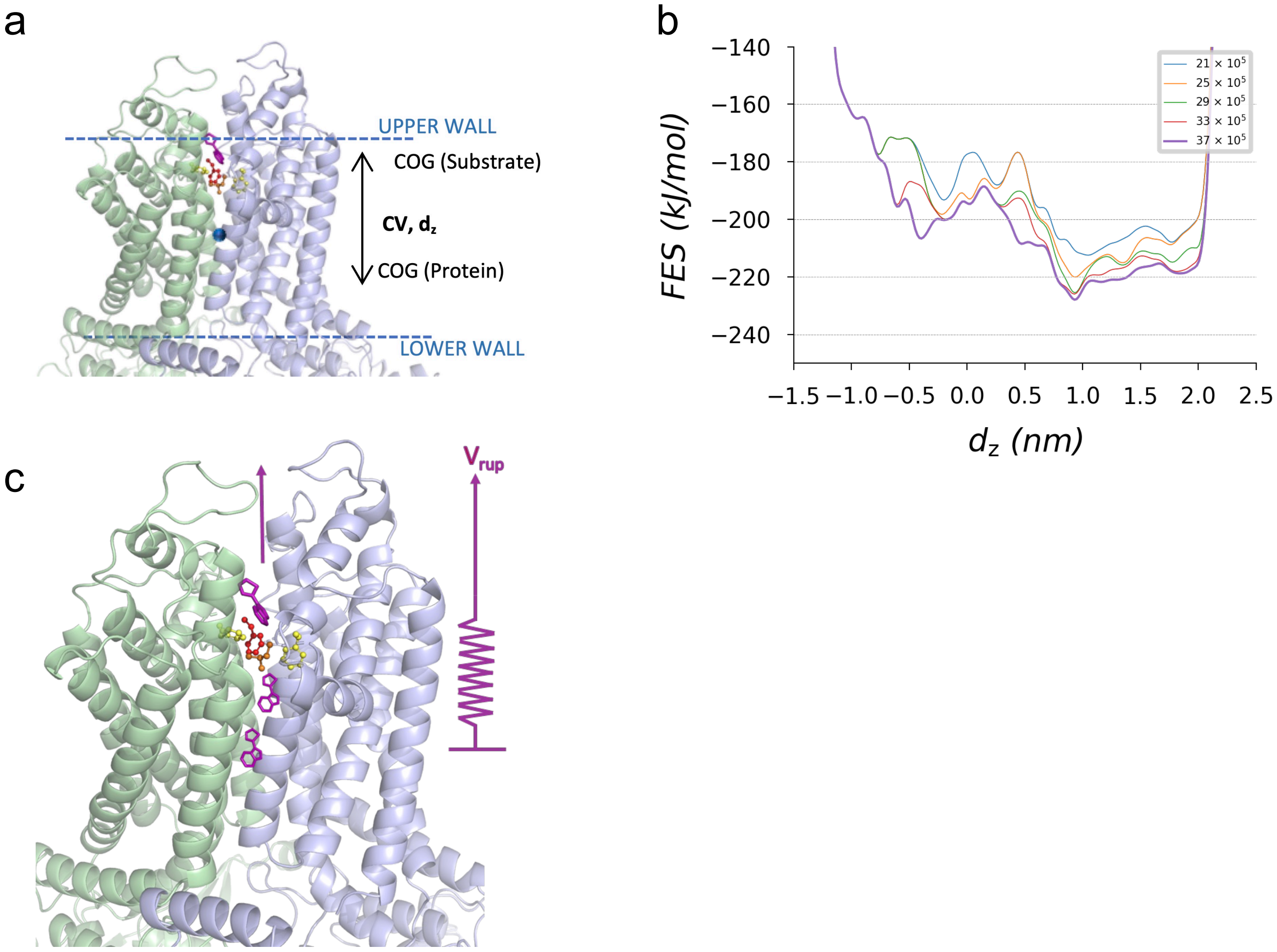

Supplementary Figure 4: Metadynamics simulation setup and scheme of ABCG36 pulling simulations

a. The reaction coordinate (collective variable, CV) for metadynamics is demonstrated using the side view of the ABCG36 structure. The z component (orthogonal to the membrane bilayer) of the distance between the center of geometry (COG; a small, blue circle, defined by the TH regions of TH2, TH5, TH8, and TH11) and the substrate defines the value of CV, limited by an upper and a lower wall to prohibit the escape of the small molecules from the translocation pathway.

b. Monitoring the convergence of metadynamics simulations. FES for Wt ABCG36 (Wt) and indicated L704 mutations of ABCG36 were calculated and plotted at indicated strides (every nth Gaussian kernel) using the *sum_hills* command of PLUMED (the simulation with Wt ABCG36 and IBA is shown as an example).

c. Force-constrained GROMACS umbrella pulling was used to displace the molecule from the central binding pocket to the extracellular space. A harmonic potential was applied (v_{rup}: the velocity at which the harmonic potential was retracted). Orange, L704; yellow, F703 and F1374; red, F1375.

Source data are provided as a Source Data file.

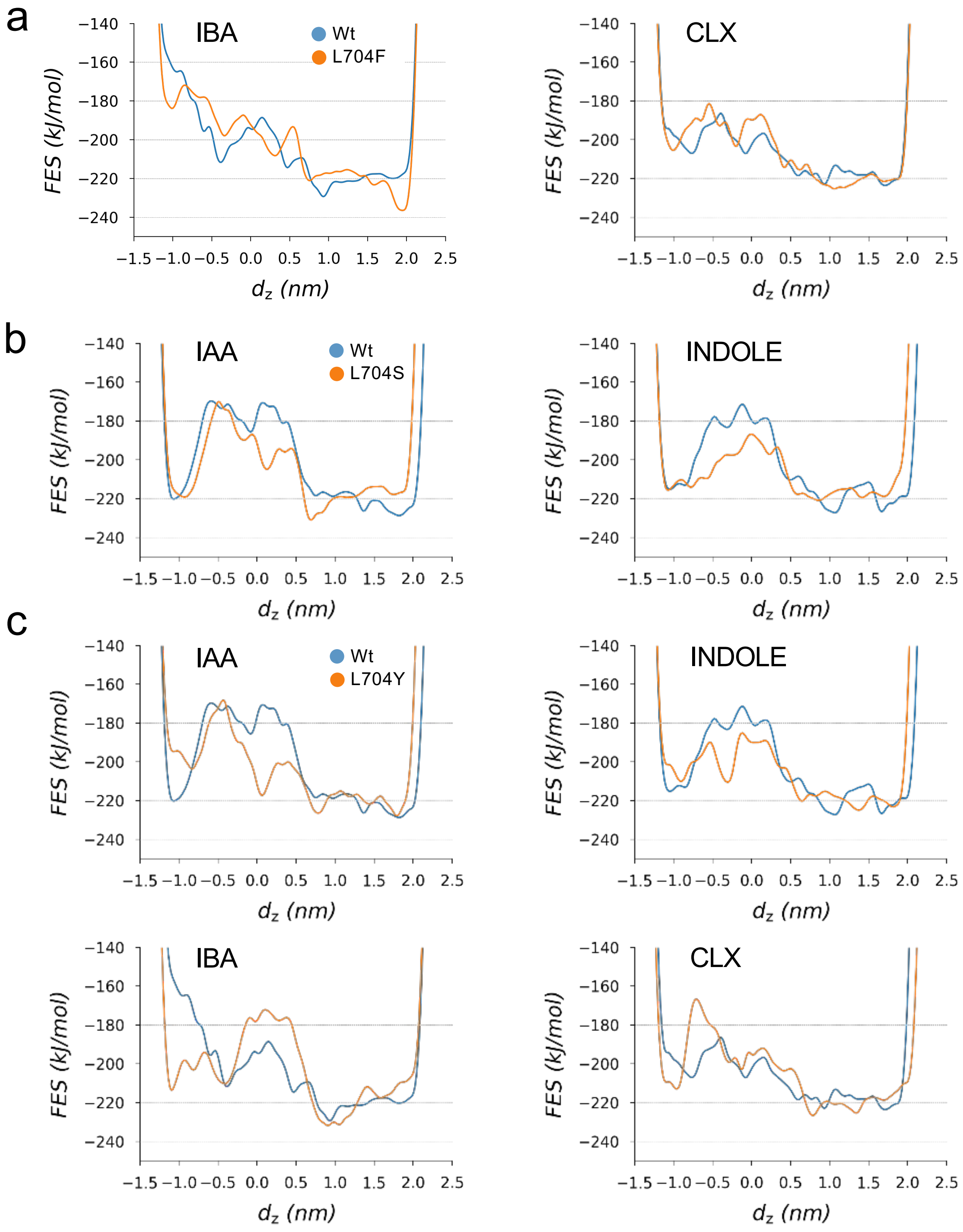

Supplementary Figure 5: Free Energy Surface (FES) calculations from metadynamics simulations.

a. The height of peaks in the FES for CLX with L704F ABCG36 was increased compared to that with Wt ABCG36. As this increase was not observed for IBA, the higher FES in the central binding pocket may hinder CLX transport by L704F ABCG36.

b. For IAA/L704S, the FES was decreased in the 0–0.5 nm region, supporting the substrate properties of IAA in this context. Similarly, the FES for Indole/L704S was decreased but in the -1–0 nm region, with a steep gradual increase toward the binding pocket.

c. The FES for both IBA/L704Y and Indole/L704Y was decreased, explaining why these molecules act as substrates for this ABCG36 mutant. Although the FES for IBA/L704Y increases in the 0–0.5 nm region, it is likely compensated by a significant decrease in the -1 to -0.5 nm region. Since the FES profiles for CLX/L704Y do not explain the substrate behavior of CLX in this context, and the FES in the valve region (1.8–2.2 nm) from simulations provided no insights into whether these residues influence substrate recognition and transport, we performed additional biased simulations (see Supplementary Figure 6).

Source data are provided as a Source Data file.

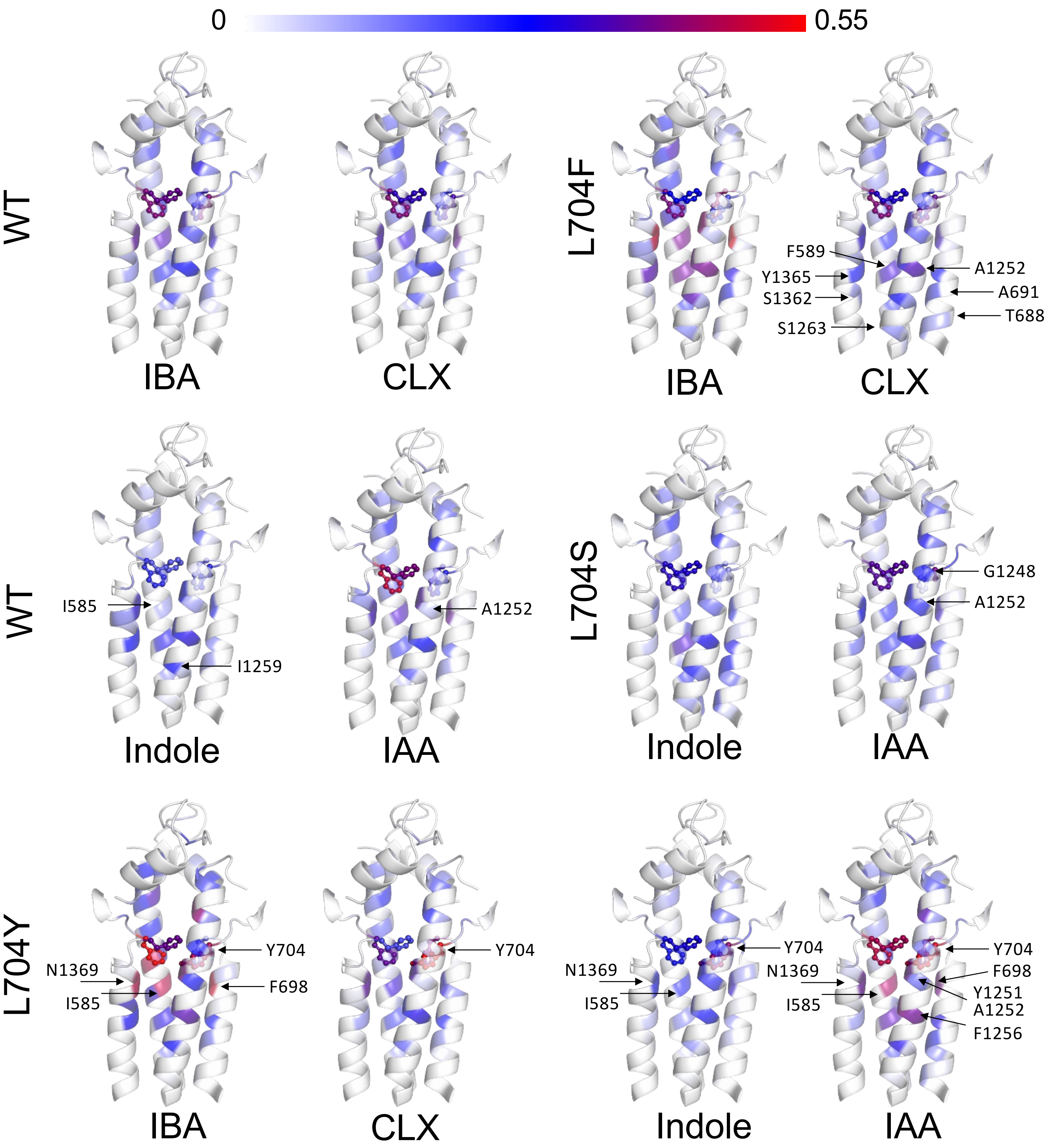

Supplementary Figure 6: Biased pulling simulations highlighted residues important for transport. Simulations revealed that CLX contacts increased in the lower region of the binding pocket for the L704F mutant when non-transported (b) compared to the wild-type (WT) ABCG36 when transported (a). This shift suggests that interactions in the upper region, near the Leu/Phe-valve, play a primary role in substrate selection and/or triggering the transport. Arrows highlight residues with contact frequencies higher than those in the Wt. This shift toward the lower part is less evident in the Indole/WT and IAA/WT non-substrate simulations (c), where arrows denote residues with increased or decreased contact frequencies compared to the Wt. For IAA/L704S (d), the shift in interactions to the upper region is minimal and it becomes prominent for IAA/L704Y (e) and Indole/L704Y (e), where these molecules act as substrates. A similar pattern is observed for IBA and CLX substrate interactions with the L704Y mutant (e). Arrows indicate residues with higher interaction frequencies compared to the WT or non-substrate setups.
Source data are provided as a Source Data file.

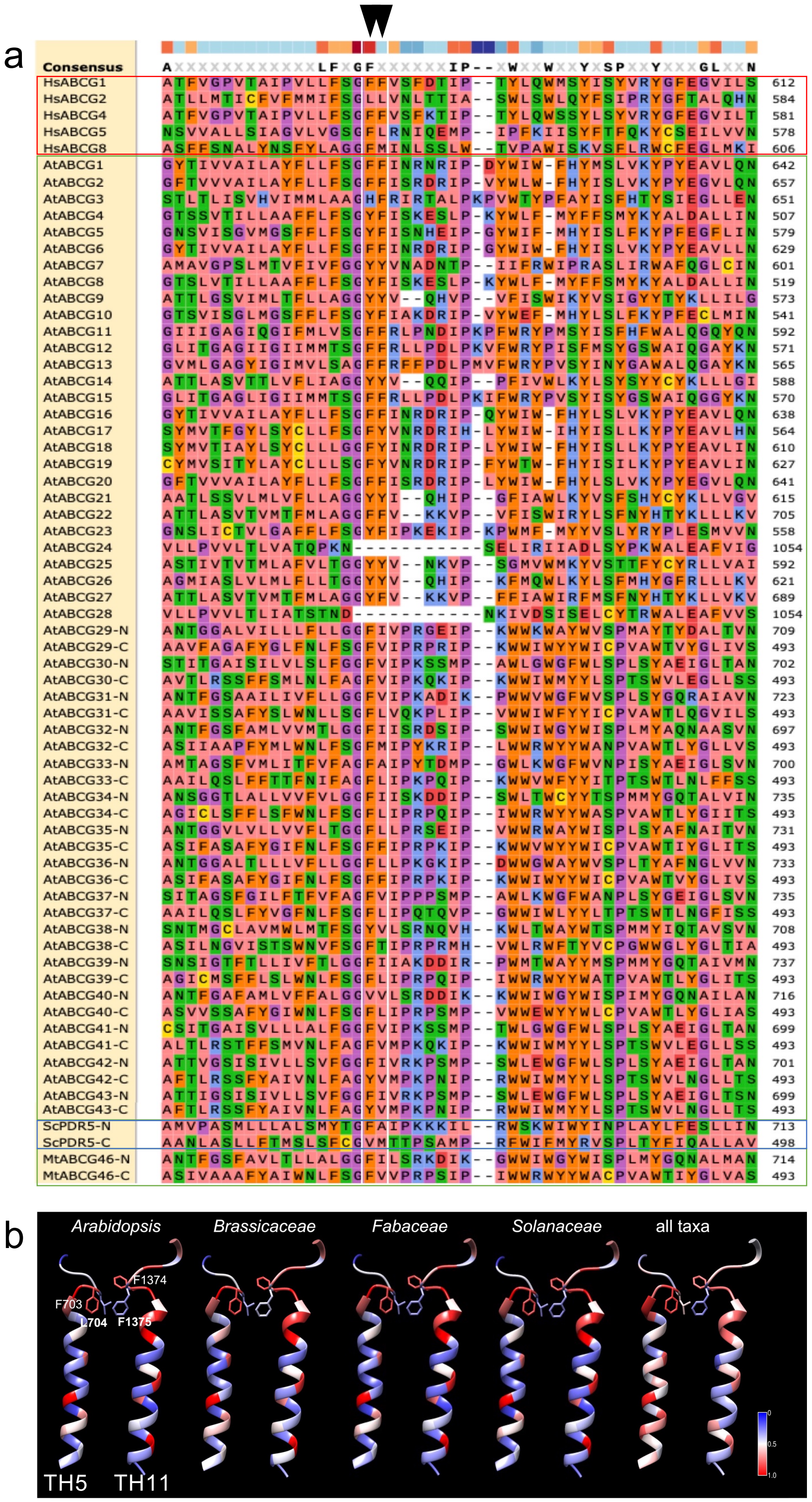

Supplementary Figure 7: Conservation of essential residues in the extracellular gate of plant PDR-type ABCGs.

a. Comparison of a proposed extracellular gate in HsABCG2 (Khunweeraphong et al., 2020; Huang et al., 2023; Ying et al., 2023) with homologous residues in Arabidopsis, human and selected other ABCGs. The position of two leucines, L554 and L555, forming the “di-leucine valve” in ABCG2 (Khunweeraphong et al., 2020), is boxed and indicated by a triangle. Human PDRS are boxedin red, yeasts ones in blue.

b. Conservation of indicated key residues of the extracellular gate in Arabidopsis. *Brassicaceae*, *Fabaceae* and *Solanaceae* families with all available sequences of plant full-size ABCG (PDR) sequences. Conservation was mapped on TH5 and TH11 and adjacent key residues corresponding to Arabidopsis F703/L704 and F1374/F1375 .
