# Supplementary material for: A key residue of the extracellular gate provides quality control contributing to ABCG substrate specificity
Source: Nat Commun. 2025 May 5;16:4177. doi: 10.1038/s41467-025-59518-3 (PMC12052975; doi:10.1038/s41467-025-59518-3)
Supplement: Supplementary file 2 — Reporting Summary [file 41467_2025_59518_MOESM2_ESM.pdf]

Corresponding author(s): Markus M. GEISLER

Last updated by author(s): 2025/02/10

Reporting Summary

Nature Portfolio wishes to improve the reproducibility of the work that we publish. This form provides structure for consistency and transparency in reporting. For further information on Nature Portfolio policies, see our Editorial Policies and the Editorial Policy Checklist.

Statistics

For all statistical analyses, confirm that the following items are present in the figure legend, table legend, main text, or Methods section.

n/a	Confirmed	
☐	☒	The exact sample size (*n*) for each experimental group/condition, given as a discrete number and unit of measurement
☐	☒	A statement on whether measurements were taken from distinct samples or whether the same sample was measured repeatedly
☐	☒	The statistical test(s) used AND whether they are one- or two-sided<br>*Only common tests should be described solely by name; describe more complex techniques in the Methods section.*
☒	☐	A description of all covariates tested
☐	☒	A description of any assumptions or corrections, such as tests of normality and adjustment for multiple comparisons
☐	☒	A full description of the statistical parameters including central tendency (e.g. means) or other basic estimates (e.g. regression coefficient) AND variation (e.g. standard deviation) or associated estimates of uncertainty (e.g. confidence intervals)
☐	☒	For null hypothesis testing, the test statistic (e.g. *F*, *t*, *r*) with confidence intervals, effect sizes, degrees of freedom and *P* value noted<br>*Give P values as exact values whenever suitable.*
☒	☐	For Bayesian analysis, information on the choice of priors and Markov chain Monte Carlo settings
☒	☐	For hierarchical and complex designs, identification of the appropriate level for tests and full reporting of outcomes
☒	☐	Estimates of effect sizes (e.g. Cohen's *d*, Pearson's *r*), indicating how they were calculated

Our web collection on statistics for biologists contains articles on many of the points above.

Software and code

Policy information about availability of computer code

Data collection	The AlphaFold ABCG36 structure was collected from https://alphafold.ebi.ac.uk/entry/Q9XIE2.
Data analysis	Multiple sequence alignment of ABCG sequences were aligned using MUSCLE. Microsoft Excel for Mac (Vers. 16.78.3) was used for analyses of evolutionary data.<br>Quantum Chemical Modeling calculations were performed with Turbomole (http://www.turbomole.com).<br>ABCG36 structure analysis was perfomed using Pymol 2.4.0a0, plotted using Matplotlib 3.7.0. For trajectory, METAD and structure and trajectory analyses, GROMACS 2022, PLUMED 2.8.1 and MDAnalysis 2.7.0 tools were used, respectively.<br>GraphPad Prism 10.4.1 for Mac was used for data analyses of transport, binding, imaging and infection studies.

For manuscripts utilizing custom algorithms or software that are central to the research but not yet described in published literature, software must be made available to editors and reviewers. We strongly encourage code deposition in a community repository (e.g. GitHub). See the Nature Portfolio guidelines for submitting code & software for further information.

Data

Policy information about availability of data

All manuscripts must include a data availability statement. This statement should provide the following information, where applicable:

- Accession codes, unique identifiers, or web links for publicly available datasets
- A description of any restrictions on data availability
- For clinical datasets or third party data, please ensure that the statement adheres to our policy

This article does not contain any original code. Requests for data should be made to and will be fulfilled by M.M. Geisler (markus.geisler@unifr.ch), provided the data will be used within the scope of the originally provided informed consent. Source data are provided with this paper. Important input and output files of MD simulations are available at Zenodo (https://doi.org/10.5281/zenodo.14743312).

Research involving human participants, their data, or biological material

Policy information about studies with human participants or human data. See also policy information about sex, gender (identity/presentation), and sexual orientation and race, ethnicity and racism.

Reporting on sex and gender	n/a
Reporting on race, ethnicity, or other socially relevant groupings	n/a
Population characteristics	n/a
Recruitment	n/a
Ethics oversight	n/a

Note that full information on the approval of the study protocol must also be provided in the manuscript.

Field-specific reporting

Please select the one below that is the best fit for your research. If you are not sure, read the appropriate sections before making your selection.

☒ Life sciences ☐ Behavioural & social sciences ☐ Ecological, evolutionary & environmental sciences

For a reference copy of the document with all sections, see nature.com/documents/nr-reporting-summary-flat.pdf

Life sciences study design

All studies must disclose on these points even when the disclosure is negative.

Sample size	The sample size for all experiments (n) is indicated in zthe figure legends.
Data exclusions	No data exclusion was performed.
Replication	All experiments were conducted as multiple, independent replicates as indicated in the figure legends.
Randomization	Randomization is not relevant to our study.
Blinding	Blinding is not relevant to our study.

Reporting for specific materials, systems and methods

We require information from authors about some types of materials, experimental systems and methods used in many studies. Here, indicate whether each material, system or method listed is relevant to your study. If you are not sure if a list item applies to your research, read the appropriate section before selecting a response.

Materials & experimental systems

n/a	Involved in the study	
☐	☒	Antibodies
☒	☐	Eukaryotic cell lines
☒	☐	Palaeontology and archaeology
☒	☐	Animals and other organisms
☒	☐	Clinical data
☒	☐	Dual use research of concern
☐	☒	Plants

Methods

n/a	Involved in the study	
☒	☐	ChIP-seq
☒	☐	Flow cytometry
☒	☐	MRI-based neuroimaging

Antibodies

Antibodies used	Anti-PDR8/ABCG36 (Agrisera product no: AS22 4846) and anti-PIP2;1 (Agrisera product no: AS22 4810) was used for Western blot analyses.
Validation	Reactivity of anti-PDR8 (https://www.agrisera.com/cgi-bin/ibutik/SkapaFaktura.pl?SkrivPDF=J&Sprak=EN&Friendly_Grupp=&funk=visa_artikel&skrivpdf=j&artnr=AS22%204846&artgrp=65&Friendly=pdr8-abc-transporter-g-family-member-36) and anti-PIP2;1 (https://www.agrisera.com/cgi-bin/ibutik/SkapaFaktura.pl?SkrivPDF=J&Sprak=EN&skrivpdf=j&funk=visa_artikel&artgrp=65&Friendly=pip21-pip22-pip23-plasma-membrane-intrinistic-protein-2-1-2-2-3&artnr=AS22%204810&Friendly_Grupp=) for Arabidopsis thaliana was confirmed by the manufacturer.

Dual use research of concern

Policy information about dual use research of concern

Hazards

Could the accidental, deliberate or reckless misuse of agents or technologies generated in the work, or the application of information presented in the manuscript, pose a threat to:

No	Yes	
☒	☐	Public health
☒	☐	National security
☒	☐	Crops and/or livestock
☒	☐	Ecosystems
☒	☐	Any other significant area

Experiments of concern

Does the work involve any of these experiments of concern:

No	Yes	
☒	☐	Demonstrate how to render a vaccine ineffective
☒	☐	Confer resistance to therapeutically useful antibiotics or antiviral agents
☒	☐	Enhance the virulence of a pathogen or render a nonpathogen virulent
☒	☐	Increase transmissibility of a pathogen
☒	☐	Alter the host range of a pathogen
☒	☐	Enable evasion of diagnostic/detection modalities
☒	☐	Enable the weaponization of a biological agent or toxin
☒	☐	Any other potentially harmful combination of experiments and agents

Plants

Seed stocks	The following Arabidopsis thaliana lines in ecotype Columbia (Col Wt) were used and obtained from the indicated sources: abcg36-4/pen3-4 (SALK_000578, Stein et al. 2006), abcg36-6/pen3-6/pdr8-115 (Strader et al. 2009). gl1 (gl1 Wt) was used as the wild-type control for abcg36-5/pen3-5 (Lu et al. 2015).
Novel plant genotypes	Mutant versions of ABCG36 were generated by QuickChange (Agilent Company, USA) site-directed mutagenesis of 35S:ABCG36 (Kim et al. 2007), 35S:ABCG36-GFP (Aryal et al. 2023) and ABCG36:ABCG36-GFP (Stein et al. 2006). Mutated plasmids were used to transform abcg36-4 by floral dipping to generate ABCG36:ABCG36L704F-GFP (abcg36-4) #1, ABCG36:ABCG36L704F-GFP (abcg36-4) #2, ABCG36:ABCG36A1357V-GFP (abcg36-4) #1, ABCG36:ABCG36A1357V-GFP (abcg36-4) #2, ABCG36:ABCG36L704F A1357V-GFP (abcg36-4) #1 and ABCG36:ABCG36L704F A1357V-GFP (abcg36-4) #2.
Authentication	Isogenic, homozygous lines for the transgene in the F3 generations were used for further analyses.
